# Pattern of sensory processing disorder in school‐aged preterm children with attention‐deficit hyperactivity disorder

**DOI:** 10.1002/pcn5.70393

**Published:** 2026-08-14

**Authors:** Marina Estima Neiva Nunes, Carla Andrea Tanuri Cardoso Caldas, Nelson Macedo Liporaci, Patricia Aparecida Zuanetti, Angela Cristina Pontes Fernandes, Carolina Augusta Portugal, Ana Paula Andrade Hamad

**Affiliations:** ^1^ Department of Neurosciences and Behavioral Sciences, Ribeirão Preto Medical School University of São Paulo Ribeirão Preto São Paulo Brazil; ^2^ Hospital das Clínicas, Ribeirão Preto Medical School University of São Paulo Ribeirão Preto São Paulo Brazil

**Keywords:** ADHD, prematurity, sensory processing, Sensory Profile‐2 assessment tool

## Abstract

**Aim:**

To evaluate sensory processing in school‐aged children born preterm with and without attention‐deficit/hyperactivity disorder (ADHD), focusing on sensory processing patterns and sensory and behavioral sections of the Sensory Profile‐2 (SP‐2).

**Methods:**

Forty‐eight school‐aged children born preterm were enrolled. The mean age was 7.9 years, and 62.5% were boys. Participants were divided into an ADHD group (*n* = 38) and a non‐ADHD group (*n* = 10) and were assessed using the SP‐2.

**Results:**

Thirty‐two participants (66.7%) showed overall sensory scores outside the typical range, of whom 28 (87.5%) had ADHD. Non‐parametric analyses revealed significantly higher scores in the Sensitivity quadrant, the Auditory section, and the Conduct and Attention behavioral sections among children with ADHD.

**Conclusion:**

Children without ADHD showed fewer sensory processing difficulties than those with ADHD, suggesting that ADHD may be associated with more pronounced sensory processing difficulties among children born preterm.

## Graphical abstract

Preterm children show high attention‐deficit/hyperactivity disorder (ADHD) prevalence and frequent sensory processing alterations. ADHD is associated with more severe impairments, particularly in sensory sensitivity, auditory processing, and behavioral domains.

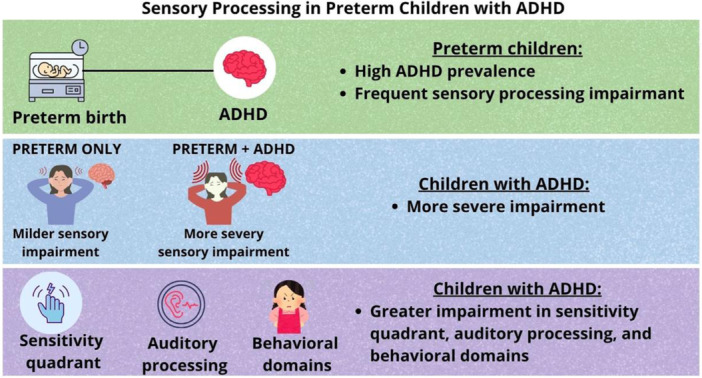

## INTRODUCTION

Sensory processing refers to the ability of the central nervous system to detect, regulate, interpret, and respond to sensory stimuli from both the body and the external environment. It results from the interaction between an individual's neurological threshold and self‐regulation. Therefore, different behavioral patterns and responses to sensory stimuli result from the combination of a high or low neurological threshold and active or passive self‐regulatory strategies.^1^, ^2^

Children's behavior is influenced by their sensory processing pattern, which can be classified into four types: (1) Registration (high threshold/passive strategy), in which the child does not readily notice sensory stimuli and does not actively seek sensory input; (2) Seeking (high threshold/active strategy), in which sensory stimuli are not easily perceived, so the child actively looks for sensory input; (3) Sensitivity (low threshold/passive strategy), in which child or adolescent are sensitive to sensory stimuli and feels discomfort when exposed to them but does not actively avoid them; and (4) Avoidance (low threshold/active strategy), in which the child or adolescent not only actively avoids sensory stimuli but also attempts to control sensory input.^1^, ^3^, ^4^

Impairments in sensory processing may affect the child's participation in sensory and motor play, therefore compromising the development of cognitive and social skills, as well as leading to detrimental performance of daily and academic activities.^5^ These limitations might result in a negative impact on relationships, self‐esteem, self‐reliance, and overall quality of life.^6^

Previous studies have shown that children with neurodevelopmental disorders respond in different ways to sensory experiences when compared to their typical peers, which might affect the child's participation in daily activities that promote learning skills.^1^, ^4^, ^5^, ^6^, ^7^, ^8^

Attention‐deficit/hyperactivity disorder (ADHD) is a neurodevelopmental disorder characterized by inattention and/or hyperactivity‐impulsivity that exceeds what is expected for a child's development.^9^ Studies have reported that 50%–60% of children with ADHD exhibit sensory processing abnormalities.^5^, ^10^, ^11^ indicating that children with ADHD not only have to face the challenges related to the core symptoms of the disorder but may also experience difficulties associated with sensory processing.

Previous research has shown that ADHD might be strongly associated with both hypo‐ and hypersensitivity, particularly about sensory sensitivity, observation, and exploration skills.^4^, ^11^ With advances in medical care, technology, and assessment methods, clearer associations between early risk factors associated with prematurity and later developmental outcomes have been established.^12^ Since then, the relationship of prematurity and/or low birth weight has been studied, initially with cerebral palsy (CP) as the main outcome.^13^ More recently, since the prevalence of CP has remained relatively stable, increasing attention has been directed toward motor impairments and subtle perceptual‐motor difficulties among this population.^3^, ^14^, ^15^, ^16^, ^17^, ^18^

Preterm birth has been associated with a 2.64‐fold increased risk of ADHD symptoms and cognitive difficulties.^19^ Previous studies have reported that 39%–82% of very preterm children (<34 weeks' gestation) exhibit atypical sensory processing patterns.^3^, ^16^ These alterations have been attributed not only to the interruption of typical intrauterine neurodevelopment but also to prolonged exposure to the neonatal intensive care unit (NICU) environment. Extended NICU hospitalization may increase exposure to sensory stimuli that differ substantially from those encountered in utero, including continuous light and noise, frequent handling, invasive procedures, respiratory support, and prolonged orotracheal intubation.^15^, ^17^, ^20^

Children with ADHD appear to process stimuli differently, either through excessive sensory input or insufficient sensory information from various sources. Sensory dysfunction at the neural level directly affects the processing of sensory information, resulting in distorted or less reliable information reaching the brain. Consequently, this may impair the formation of accurate mental representations of the environment, thereby affecting cortical sensory processing regions involved in cognitive functions.^21^

At a broader level, sensory processing dysfunctions result in long‐term functional difficulties across the lifespan, from early infancy to adulthood, due to a reduction in the frequency, duration, or complexity of adaptive responses. These difficulties may ultimately interfere with an individual's confidence, self‐esteem, and motor skills. Therefore, ADHD has been associated with sensory processing difficulties, although the underlying pathophysiological mechanisms remain poorly understood.^5^, ^20^

Although prematurity^3^, ^16^, ^17^, ^20^, ^22^, ^23^, ^24^, ^25^, ^26^, ^27^ and ADHD^2^, ^3^, ^4^, ^7^, ^10^, ^11^, ^28^, ^29^, ^30^ have been individually associated with sensory processing difficulties, these conditions may interact through partially overlapping mechanisms that influence sensory modulation and behavioral responses to sensory stimuli. Consequently, the coexistence of prematurity and ADHD may contribute to greater variability and potentially more pronounced sensory processing difficulties than either condition alone. However, it remains unclear whether sensory processing patterns differ between preterm children with and without ADHD. Understanding this relationship may provide insights into the heterogeneity of sensory processing outcomes among preterm children.

We hypothesized that preterm children with ADHD would present more pronounced sensory processing difficulties than preterm children without ADHD. Therefore, the primary aim of this study was to compare sensory processing characteristics between school‐aged children born preterm with and without ADHD using the Sensory Profile‐2 (SP‐2).^31^, ^32^ As a secondary exploratory analysis, we investigated potential associations between neonatal factors and sensory processing outcomes.

## MATERIALS AND METHODS

This cross‐sectional study included a cohort of preterm children born before 35 weeks of gestation between 2012 and 2016 at a university hospital and subsequently followed in the institution's neonatal follow‐up outpatient clinic. According to the hospital protocol, all infants born before 35 weeks of gestation are initially enrolled in the neonatal follow‐up program after hospital discharge. However, preterm infants with major neonatal neurological complications or conditions associated with a high risk of severe neurodevelopmental impairment are routinely referred to specialized pediatric neurology services. Consequently, the neonatal follow‐up clinic primarily monitors preterm children without major neonatal neurological complications requiring specialized pediatric neurology care.

To identify the study population, hospital electronic medical records were screened to identify all children born between 2012 and 2016 with a gestational age < 35 weeks who had reached school age at the time of the study. This initial screening identified 133 children, whose eligibility was subsequently determined according to the predefined inclusion and exclusion criteria.

Pre‐ and perinatal data were obtained from electronic medical records. Parents or caregivers completed the following instruments: the Swanson, Nolan, and Pelham‐IV (SNAP‐IV) questionnaire,^33^ the Child SP‐2 – Brazilian version,^32^ the Child Behavior Checklist (CBCL),^34^ and the Brazilian Economic Classification Criteria (ABEP).^35^ The SP‐2^32^ was used to characterize sensory processing patterns in everyday contexts, while the CBCL^34^ was used to assess emotional and behavioral functioning, including the presence of internalizing and externalizing symptoms. All children underwent comprehensive clinical, neurological, and neuropsychological assessments.

Clinical assessments were conducted by the same multidisciplinary team, consisting of a pediatric neurologist, a psychologist, and a speech‐language therapist. Inter‐rater reliability was not formally assessed because the diagnostic evaluation was conducted by consensus within the same multidisciplinary team. ADHD diagnosis was established according to the Diagnostic and Statistical Manual of Mental Disorders, Fifth Edition (DSM‐5)^9^ criteria through a structured developmental and behavioral interview with parents or caregivers. Information regarding symptom onset, persistence, functional impairment, and symptom presentation across multiple settings was obtained during the clinical interview and complemented by SNAP‐IV^33^ questionnaires completed by parents and teachers. All participants underwent a neurological examination performed by the pediatric neurologist, and only children with normal neurological findings were included in the study.

The SNAP‐IV^33^ was used as a supportive instrument during the diagnostic process rather than as a stand‐alone diagnostic tool. All participants also underwent a neuropsychological assessment, including the Wechsler Intelligence Scale for Children – Fourth Edition (WISC‐IV), Portuguese version, and the Psychological Battery for Attention Assessment (BPA). Neuropsychological testing was used to characterize cognitive and attentional functioning to support the clinical evaluation. Children with a full‐scale IQ < 70, congenital abnormalities, CP, epilepsy, intellectual disability (ID), autism spectrum disorder (ASD), or communication disorders were excluded. None of the participants were receiving pharmacological treatment for ADHD at the time of the sensory, behavioral, and neuropsychological assessments.

The study was approved by the Ethics Committee under protocol number 43962621.9.0000.5440, and parental written informed consent was obtained for all participants.

### The Child SP‐2 questionnaire

Sensory processing was assessed using the Brazilian version of the Child SP‐2,^32^ a standardized caregiver‐report questionnaire designed for children aged 3–14 years. The instrument consists of 86 items rated on a 5‐point Likert scale ranging from 1 (Almost Never) to 5 (Almost Always), with an additional response option of 0 (Does Not Apply). Higher scores indicate a greater frequency of behaviors associated with the respective sensory pattern or domain. The Brazilian version of the SP‐2 has undergone cross‐cultural adaptation and demonstrates adequate psychometric properties for use in Brazilian children.

The SP‐2 is based on Dunn's Sensory Processing Framework and provides scores across four sensory processing quadrants (Seeking, Avoidance, Sensitivity, and Registration), six sensory sections (Auditory, Visual, Touch, Movement, Body Position, and Oral), and three behavioral sections (Conduct, Social Emotional, and Attention). Raw scores are calculated for each quadrant and section and are subsequently compared with age‐based normative data provided in the instrument manual.

Raw scores obtained for each quadrant and section are converted into performance categories using age‐based normative data. These categories indicate how a child's sensory processing patterns compare with those of peers in the normative sample and are derived from score distributions based on a bell‐shaped curve. According to the SP‐2 classification system, performance is categorized as Much Less Than Others, Less Than Others, Just Like the Majority of Others, More Than Others, or Much More Than Others.

**Figure 1:**
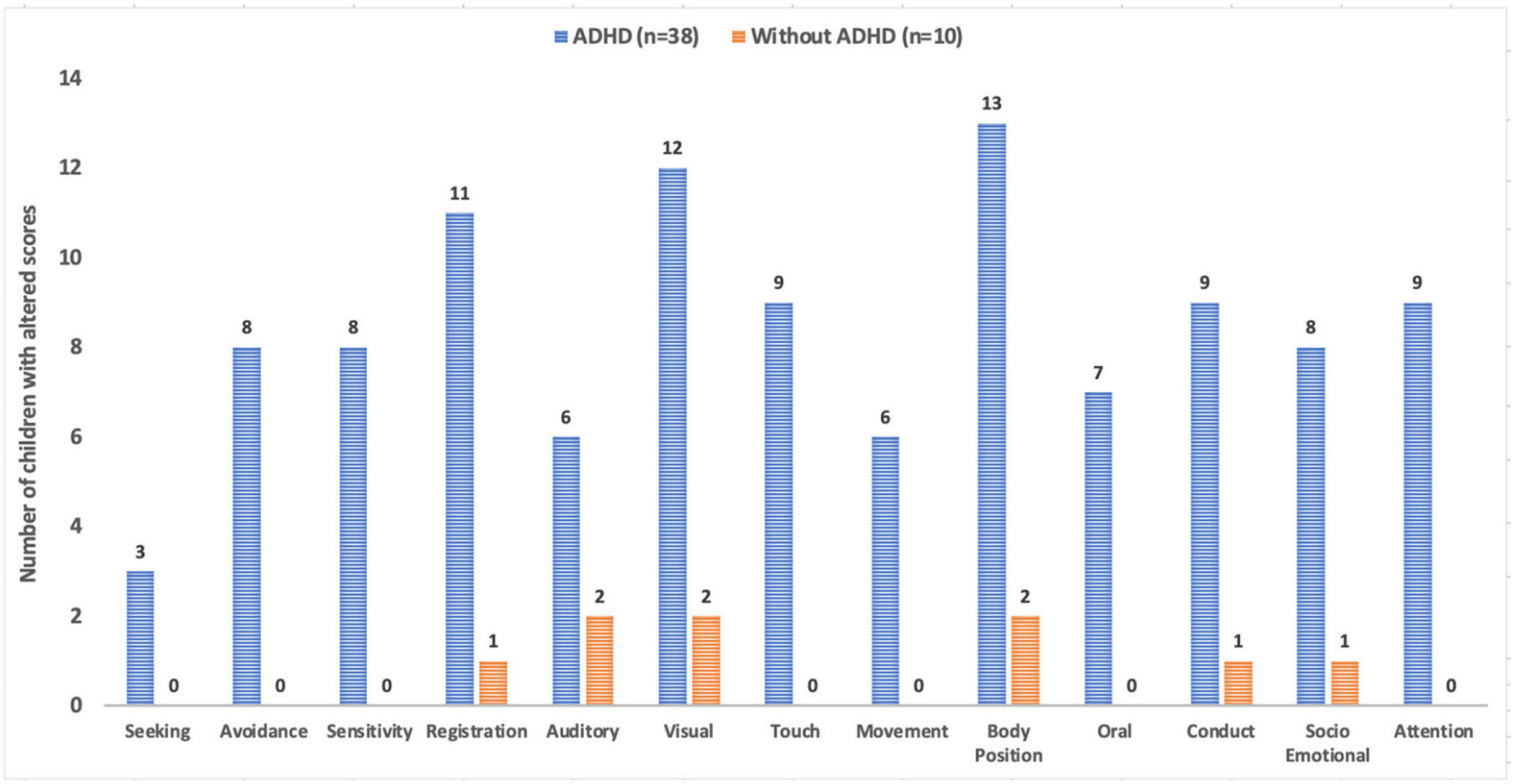
Number of children with altered scores in Sensory Profile‐2 (SP‐2) quadrants, sensory sections, and behavioral sections among preterm children with and without attention‐deficit/hyperactivity disorder (ADHD). Data are presented as absolute frequencies.

For this study, scores classified as Much Less Than Others or Much More Than Others were considered indicative of atypical sensory processing patterns. According to the SP‐2 classification system, these categories correspond to scores falling two or more standard deviations below or above the normative mean, respectively. These classifications were used to calculate the proportion of participants with atypical sensory processing patterns as presented in Figure 1.

In addition to the categorical classification, continuous raw scores were analyzed to compare sensory processing performance between groups. These raw score distributions are presented in Table 2, where higher scores indicate a greater frequency of behaviors associated with the corresponding sensory quadrant, sensory section, or behavioral section.

### The CBCL

Standardized instrument used to identify emotional and behavioral problems in children and adolescents.^34^ It assesses a broad range of domains, including affective problems, anxiety problems, somatic complaints, ADHD, oppositional defiant disorder, conduct disorder, obsessive–compulsive disorder, and post‐traumatic stress disorder. The instrument also provides composite scores for Internalizing Problems and Externalizing Problems. Designed for children and adolescents aged 6–18 years, the CBCL 6–18 consists of 113 items completed by parents or caregivers, each rated on a 3‐point Likert scale (0 = not present, 1 = sometimes present, and 2 = often present). *T* scores are derived from the raw scores, with values above 63 indicating clinically significant problems and scores between 60 and 63 considered within the borderline clinical range.^34^

### The SNAP‐IV questionnaire

Instrument^33^ used to screen for and assess the frequency of symptoms of inattention, hyperactivity, and impulsivity based on the criteria of the Diagnostic and Statistical Manual of Mental Disorders, Fourth Edition (DSM‐IV). The Brazilian Portuguese version of the SNAP‐IV, developed as part of the Multimodal Treatment Assessment of Children with ADHD (MTA) study, was used in the present study. This version includes 18 ADHD symptoms, comprising 9 inattentive and 9 hyperactive/impulsive symptoms.^36^ Although the SNAP‐IV is based on DSM‐IV criteria, it was used solely as a screening and supportive instrument, whereas the final ADHD diagnosis was established according to DSM‐5 criteria.

### ABEP, 2020

Socioeconomic status was assessed using the ABEP,^35^ which is based on a combination of variables evaluated through a standardized questionnaire. The instrument considers household ownership of consumer goods, the educational level of the head of the household, and access to public services, such as piped water and sewage. Based on the total score, households are classified into the following socioeconomic strata: A, B1, B2, C1, C2, and DE. The estimated average monthly household income for each stratum is as follows: Class A, R$22,749.24; B1, R$10,788.56; B2, R$5,721.72; C1, R$3,194.33; C2, R$1,894.95; and DE, R$862.41. For the purposes of this study, Classes A and B were grouped, as were Classes C and DE.

### Statistical analysis

Study data were collected and managed using REDCap (Research Electronic Data Capture) electronic data capture tools hosted at the University of São Paulo.

Continuous variables were presented as mean ± standard deviation or median (range or interquartile range), according to data distribution. Categorical variables were presented as frequencies and percentages.

Comparisons of continuous variables between children with and without ADHD were performed using the Mann–Whitney *U* test. Associations between categorical variables were examined using the chi‐square test or Fisher's exact test, as appropriate. Statistical significance was set at *p* ≤ 0.05. No correlation analyses were performed because the exploratory analyses focused on categorical associations.

Additional exploratory analyses were conducted to investigate potential associations between atypical sensory processing patterns and selected clinical variables, including ADHD presentations, internalizing and externalizing behavioral problems assessed using the CBCL, the need for orotracheal intubation during the neonatal period, abnormal transfontanellar ultrasound findings identified during the neonatal follow‐up protocol, and socioeconomic variables. For significant associations between categorical variables, odds ratios (ORs) and 95% confidence intervals (95% CIs) were calculated from contingency tables.

## RESULTS

A total of 133 preterm children were initially selected, of whom 46 met the exclusion criteria. Of the remaining 87 children, 18 could not be contacted despite attempts by telephone or mail, 15 declined to participate, and 2 did not complete the study protocol. Among the 52 participants who completed the initial assessment, 4 were subsequently excluded for not meeting the inclusion criteria: 3 met criteria for ASD Level 1, and 1 had ID. Ultimately, 48 children were included in the study. Participants were between 7 and 9 years of age, with a slight predominance of males (62.5%). No additional abnormalities were identified during the clinical or basic neurological examination.

The demographic and clinical characteristics of the study population are presented in Table 1.

**Table 1:** Sociodemographic and clinical data.

Characteristics	Total (*n* = 48)	ADHD (*n* = 38)	No‐ADHD (*n* = 10)	*p* ≤ 0.05
*Demographics*				
Age at examination (years)	7.9 (0.8)	7.7 (0.8)	8.4 (0.7)	0.49
Male sex	30 (62.5)	23 (60.5)	7 (70.0)	0.58
Gestational age (weeks)	30.3 (2.4)	30.4 (2.2)	30.4 (2.59)	0.33
Birth weight (g)	1249.0 (312.6)	1216.0 (340.7)	1247.5 (434.7)	0.33
*Perinatal risk factors*				
Maternal age at birth (years)	28.2 (5.9)	28.3 (5.9)	28.0 (6.1)	0.10
Hypertension	13 (27.1)	10 (26.3)	3 (30.0)	0.8
Others (diabetes mellitus, hypothyroidism)	5 (10.4)	4 (10.5)	1 (10.0)	0.96
Substance use				
Smoking	9 (18.8)	8 (21.1)	1 (10.0)	0.43
Alcohol consumption	5 (10.4)	4 (10.5)	1 (10.0)	0.96
Illicit drugs	3 (6.3)	2 (5.3)	1 (10.0)	0.58
*Socioeconomic risk factors*				
Family socioeconomic score				
Socioeconomic Class A/B	21 (43.8)	16 (42.1)	5 (50.0)	0.65
Socioeconomic Class C/DE	27 (56.3)	22 (57.9)	5 (50.0)	0.3
*Parental education level*				
Completed secondary education	39 (81.2)	32 (84.2)	7 (70.0)	0.65
Incomplete secondary education	9 (18.8)	6 (15.8)	3 (30.0)	0.30
*Neonatal risk factors*				
Abnormal transfontanellar ultrasound findings	20 (41.7)	14 (38.4)	6 (60.0)	0.19
Orotracheal intubation	13 (27.1)	10 (26.3)	3 (30)	0.82
NICU length of stay in days^a^	11.7 (13.6) 8 (0‐74)	12.7(14.7) 11 (0‐74)	7.7 (6.9) 5 (3‐24)	0.4

*Note*: Data are expressed in mean (standard deviation) or *n* (%).

Abbreviations: ADHD, attention‐deficit/hyperactivity disorder; NICU, neonatal intensive care unit.

^a^ Mean (SD); median (range).

ADHD criteria were met by 38 of the 48 children included in the study (79.2%). Regarding ADHD presentations, 22 children (57.9%) were classified as predominantly inattentive, 2 (5.3%) as predominantly hyperactive/impulsive, and 14 (36.8%) as having the combined presentation.

Thirty‐two participants (66.7%) exhibited atypical sensory processing patterns on the SP‐2 (Figure 1). The Registration quadrant was the most frequently affected, followed by the Avoidance, Sensitivity, and Seeking quadrants. Among the sensory sections, Body Position was the most frequently altered, whereas Conduct was the most frequently altered behavioral section.

Children with ADHD, 28 of 38 (73.7%), exhibited an atypical sensory processing profile. In this subgroup, the Registration quadrant was the most frequently affected (*n* = 11), followed by the Avoidance and Sensitivity quadrants (*n* = 8 each), whereas the Seeking quadrant was the least frequently affected (*n* = 3). The Body Position section demonstrated the highest frequency of atypical sensory scores (*n* = 13), while Conduct and Attention were the behavioral sections most frequently classified as atypical (*n* = 9 each) (Figure 1).

Comparison of SP‐2 scores between preterm children with ADHD (*n* = 38) and those without ADHD (*n* = 10) using the Mann–Whitney *U* test revealed significant differences in the Sensitivity quadrant (*p* = 0.03), Auditory section (*p* = 0.002), Conduct section (*p* = 0.05), and Attention section (*p* = 0.01). No significant differences were identified in the remaining quadrants or sections, including the Social Emotional section (Table 2).

**Table 2:** Altered performance scores in Sensory Profile‐2 in preterm children.

Category	All children = 48	With ADHD = 38	Without ADHD = 10	*p* ≤ 0.05
*Seeking*				No
Median (min–max)	35 (0–83)	44 (0–83)	24 (14–44)	
Mean (SD)	36.33 ± 18.7	38.92 ± 19.7	26.50 ± 9.4	
*Avoidance*				No
Median (min–max)	34.5 (3–77)	38 (3–77)	26 (12–54)	
Mean (SD)	34.79 ± 18.8	36.53 ± 19.9	28.20 ± 12.7	
*Sensitivity*				*p* = 0.03*
Median (min–max)	33 (2–75)	35 (2–75)	21.5 (13–40)	
Mean (SD)	31.9 ± 16,8	34.29 ± 17.8	22.80 ± 8.1	
*Registration*				No
Median (min–max)	25 (0–74)	31.5 (2–74)	22 (0–48)	
Mean (SD)	28.94 ± 19.4	30.66 ± 20.5	22.40 ± 13.5	
*Auditory*				*p* = 0.002*
Median (min–max)	17 (0–36)	21 (0–36)	9 (1–17)	
Mean (SD)	16.69 ± 9.5	18.87 ± 9.3	8.40 ± 4.6	
*Visual*				No
Median (min–max)	8.5 (0–23)	9.5 (0–23)	6.5 (0–13)	
Mean (SD)	8.9 ± 6.13	9.53 ± 6.5	6.40 ± 3.4	
*Touch*				No
Median (min–max)	12.5 (0–49)	15 (0–49)	12 (2–22)	
Mean (SD)	13.8 ± 10.2	14.4 ± 11.2	11.8 ± 5	
*Movement*				No
Median (min–max)	15 (0–36)	16 (0–36)	12 (4–17)	
Mean (SD)	15.4 ± 7.2	16.37 ± 7.5	12.00 ± 4.3	
*Body position*				No
Median (min–max)	8 (0–26)	8 (0–26)	8 (0–20)	
Mean (SD)	8.3 ± 7.2	8.47 ± 7.7	7.6 ± 5.2	
*Oral*				No
Median (min–max)	13 (0–40)	14.5 (0–40)	12 (10–29)	
Mean (SD)	15.8 ± 10.7	15.8 ± 11.5	15.60 ± 7.7	
*Conduct*				*p* = 0.05*
Median (min–max)	15 (0–39)	16.5 (0–39)	10.5 (0–21)	
Mean (SD)	16.6 ± 10,2	18.03 ± 10.7	11 ± 5.85	
*Social emotional*				No
Median (min–max)	22.5 (0–52)	25 (0–52)	18.5 (13–42)	
Mean (SD)	24.5 ± 14.5	25.13 ± 15.5	22.20 ± 9.7	
*Attention*				*p* = 0.01*
Median (min–max)	16.5 (0–47)	20.5 (0–47)	10.5 (2–28)	
Mean (SD)	18.8 ± 12.02	20.8 ± 12.31	11.1 ± 7.047	

*Note*: Descriptive statistics (median, minimum, and maximum values; mean ± standard deviation) of altered result scores for each quadrant and/or section of Sensory Profile‐2 (SP‐2) among all children, and children with or without ADHD.

Abbreviation: ADHD, attention‐deficit/hyperactivity disorder.

* *p* values obtained using the Mann–Whitney *U* test.

Exploratory analyses were conducted to examine potential associations between SP‐2 outcomes and selected perinatal and neonatal variables, including the need for orotracheal intubation, abnormal neonatal cranial ultrasound findings, and duration of NICU hospitalization. Only orotracheal intubation was significantly associated with atypical scores in the Movement and Social Emotional sections. Children who underwent intubation exhibited a higher frequency of atypical Movement section scores compared with those who did not require intubation (30.8% vs. 5.7%; OR = 7.33, 95% CI: 1.15–46.66; *p* = 0.04), as well as atypical Social Emotional section scores (38.5% vs. 11.4%; OR = 4.84, 95% CI: 1.05–22.31; *p* = 0.05).

CBCL results indicated that 14.6% (*n* = 7) of the participants were classified within the Internalizing Problems range, whereas 16.7% (*n* = 8) were classified within the Externalizing Problems range. All participants with Externalizing Problems belonged to the ADHD group, corresponding to a frequency of 21.1% (*n* = 8). In addition, Internalizing Problems were more frequent among children with ADHD than among those without ADHD (15.8%, *n* = 6 vs. 10.0%, *n* = 1). Exploratory analyses examining potential associations between Internalizing and Externalizing Problems and SP‐2 outcomes, as well as other study variables, did not identify any statistically significant associations.

## DISCUSSION

The present study evaluated 48 school‐aged children born preterm who had been followed in a neonatal follow‐up program at a tertiary university hospital. Of these, 38 children (79.2%) met DSM‐5 criteria for ADHD. The demographic, neonatal, and socioeconomic characteristics of the sample are summarized in Table 1, and no significant differences were identified between groups for most of the neonatal and socioeconomic variables evaluated. The high prevalence of ADHD observed in this sample may be partly explained by the characteristics of the study population, as participants were recruited from a tertiary neonatal follow‐up program. Consequently, this cohort may have included a higher proportion of children with neurodevelopmental vulnerabilities, which could have contributed to the elevated frequency of ADHD observed in the present study.

Approximately 66.7% of preterm children presented atypical sensory processing patterns. These findings are consistent with previous reports suggesting that children born preterm frequently experience sensory processing difficulties.^15^, ^16^, ^17^, ^20^, ^27^ The need for orotracheal intubation was associated with increased odds of atypical scores in the Movement section (OR = 7.33; 95% CI 1.15–46.66) and the Social Emotional section (OR = 4.84; 95% CI 1.05–22.31). Similar findings were reported by Crozier et al.,^16^ who investigated the association between neonatal risk factors and sensory processing patterns in children born very preterm. In that cohort, nearly half of the children presented atypical sensory processing patterns, and those who required intubation had lower APGAR scores, while infants with longer NICU stays were more likely to demonstrate atypical sensory processing patterns.^16^

Preterm birth has been associated with substantial alterations in early sensory experiences, particularly among infants requiring admission to the NICU. In this environment, newborns are exposed to sensory stimuli that differ markedly from those encountered in utero, including increased auditory, visual, tactile, and painful stimuli related to medical care and monitoring procedures.^3^, ^20^, ^37^ Previous studies have suggested that these early sensory experiences may contribute to differences in sensory processing outcomes later in development. Although NICU length of stay was not significantly associated with sensory processing outcomes in the current sample, children with ADHD had numerically longer NICU stays than those without ADHD (Table 1). This finding, together with the association between orotracheal intubation and atypical sensory processing patterns, may reflect greater neonatal clinical complexity rather than a specific effect of any individual neonatal exposure. Accordingly, these associations should be interpreted with caution. Furthermore, given the cross‐sectional design of the study, causal relationships cannot be established.^3^, ^16^, ^17^, ^38^

Sensory processing difficulties may represent an important functional challenge for children, as they have been associated with behavioral problems, immature social skills, and impairments in both fine and gross motor abilities. These difficulties may negatively affect performance in daily activities, interfere with adaptive social functioning, and contribute to reduced participation in academic and social contexts.^2^, ^20^, ^38^

Atypical sensory processing patterns were identified in 28 of the 38 children with ADHD (73.7%). Atypical scores were identified across all four SP‐2 quadrants. In contrast, only one child without ADHD presented an atypical score in the Registration quadrant (Figure 1). Similar findings have been reported by Rani et al.^5^ and Fabio et al.^21^ Rani et al. investigated sensory processing difficulties in children and adolescents with ADHD and reported atypical sensory processing patterns across all sensory quadrants when compared with typically developing peers, with particularly pronounced differences in the Seeking and Sensitivity quadrants.^5^ Likewise, Fabio et al., in a study including 69 adolescents with ADHD and 69 controls, identified atypical scores across all four sensory quadrants and multiple sensory sections, including Movement, Vision, Touch, and Hearing.^21^ Taken together, these findings are consistent with previous reports suggesting an association between ADHD symptoms and sensory processing difficulties across multiple sensory domains.^2^, ^5^, ^10^, ^21^, ^30^

Several studies have attempted to explain the association between ADHD symptoms and sensory processing difficulties. Proposed mechanisms include alterations in frontostriatal, frontocerebellar, and basal ganglia networks, which may affect the integration and modulation of sensory information. Some authors have suggested that sensory processing difficulties may interfere with the acquisition and automatization of skills, increasing the cognitive demands required to perform everyday activities. Consequently, sensory processing difficulties may increase the cognitive demands required for everyday activities, potentially contributing to challenges involving attention, impulse regulation, and other cognitive functions in individuals with ADHD.^21^, ^39^ Consistent with this perspective, previous studies have reported that sensory processing difficulties may negatively affect participation and performance across school, home, leisure, and community settings among children with ADHD.^1^, ^6^

Comparison of sensory processing patterns between preterm children with and without ADHD revealed a higher frequency of atypical sensory processing patterns across all four SP‐2 quadrants in children with ADHD (Figure 1), consistent with previous reports.^2^, ^5^, ^10^, ^30^ In contrast, only one child in the non‐ADHD group presented an altered pattern in the Registration quadrant.

Analysis of the raw SP‐2 scores (Table 2) revealed significantly higher scores in the Sensitivity quadrant among preterm children with ADHD than among those without ADHD. Higher scores in this quadrant indicate greater responsiveness to sensory stimuli and may be associated with increased discomfort or reactivity to everyday sensory experiences, such as sounds, lights, or textures.^30^, ^40^ Consistent with these findings, Shimizu and Miranda,^30^ in a literature review, reported significant differences in sensory processing patterns between children with ADHD and typically developing controls, highlighting difficulties in sensory modulation and behavioral responses to everyday stimuli. Likewise, Yochman et al.^40^ described increased sensory sensitivity in children with ADHD, which may contribute to challenges in academic and social functioning.

Participants with ADHD presented altered sensory processing patterns across all sensory and behavioral sections evaluated, whereas children without ADHD showed alterations in only three sensory sections and two behavioral sections. Among the sensory sections, the highest frequency of atypical patterns was observed in the Body Position section (*n* = 13), followed by the Visual section (*n* = 12) (Figure 1). Because motor function was not formally assessed, it was not possible to distinguish whether the atypical Body Position findings primarily reflected ADHD‐related sensory processing differences, co‐occurring motor difficulties, or a combination of both.

Beyond the frequency of atypical sensory processing patterns, analysis of the raw SP‐2 scores (Table 2) revealed significantly higher scores in the Auditory section among children with ADHD. Although the present study did not investigate the neural mechanisms underlying these differences, evidence from neuroimaging studies may help contextualize our findings. Neuroimaging studies have suggested that auditory sensory difficulties in ADHD may be related to alterations in neural networks involved in sensory integration and attentional modulation. Reduced functional connectivity between prefrontal and sensory regions, including the auditory cortex, has been proposed as one potential mechanism underlying less efficient filtering of irrelevant sensory stimuli. In addition, alterations in frontostriatal and frontoparietal networks, together with differences in dopaminergic signaling, have been implicated in atypical responses to sensory information in individuals with ADHD.^39^, ^41^ Collectively, these findings provide a plausible neurobiological framework for understanding the auditory sensory differences identified in our sample.

In addition to the differences identified in the Sensitivity quadrant and Auditory section, it is important to consider the potential functional implications of sensory processing difficulties across development. Sensory processing difficulties have been associated with functional challenges from early childhood to adulthood, including reductions in the frequency, duration, and complexity of adaptive responses. Alterations involving sensory sensitivity, auditory processing, attention, and behavioral regulation may interfere with everyday functioning and participation across home, school, and social environments. These difficulties may negatively affect self‐confidence, self‐esteem, and motor performance.^5^, ^20^

Despite the potential functional implications of sensory processing difficulties, the frequency of Internalizing Problems and Externalizing Problems identified in the present sample was relatively low. This finding should be interpreted with caution, particularly given the limited sample size and the small number of participants classified within the clinical range of the CBCL. Further studies with larger samples are needed to better characterize the relationship between atypical sensory processing patterns and behavioral problems in school‐aged children born preterm, with and without ADHD.

Consistent with previous reports, no significant associations were identified between ADHD presentations and sensory processing patterns assessed by the SP‐2.^5^, ^6^, ^8^, ^27^ Future studies with larger samples, particularly those including a greater number of children with the hyperactive/impulsive presentation, are warranted to further investigate whether sensory processing patterns differ across ADHD presentations.

Limitations: This study has several limitations that should be considered when interpreting the findings. First, all participants were born preterm, precluding evaluation of the independent contribution of prematurity to sensory processing outcomes. Second, the relatively small and unbalanced sample size, particularly in the non‐ADHD group, may have reduced the statistical power and precision of between‐group comparisons, increasing the risk of Type II error. Information on eligible participants who were not included in the study was unavailable, precluding comparisons between included and non‐included participants. Consequently, the potential impact of selection bias cannot be excluded. Third, no term‐born control group was included, limiting the generalizability of the findings. In addition, multiple comparisons were performed across SP‐2 quadrants and sections without adjustment for multiplicity, which may have increased the risk of Type I error. Therefore, the statistically significant differences identified in the SP‐2 should be regarded as exploratory and interpreted with appropriate caution until confirmed in larger, independent cohorts.

An additional limitation relates to the potential influence of motor difficulties on sensory processing outcomes, as motor impairments are common among children born preterm. Consequently, the high frequency of atypical patterns identified in the Body Position section of the SP‐2 may partially reflect the presence of co‐occurring motor difficulties, such as developmental coordination disorder (DCD). Future studies are needed to disentangle the independent contributions of ADHD and motor difficulties, particularly DCD, to sensory processing outcomes in children born preterm.

Participants were recruited from a single neonatal follow‐up program at a tertiary university hospital, which may limit the generalizability of these findings to the broader population of children born preterm. Inter‐rater reliability was not formally assessed because the diagnostic evaluation was conducted by consensus within the same multidisciplinary team; this should be considered when interpreting the diagnostic classification. Nevertheless, atypical sensory processing patterns were more frequently identified among preterm children with ADHD than among those without ADHD. To our knowledge, few studies have specifically examined sensory processing patterns in children born preterm according to ADHD status, and evidence regarding this population remains limited. These findings contribute to a better understanding of sensory processing characteristics in this clinically relevant population and may help inform future studies investigating the interplay between prematurity, ADHD, and sensory processing.

## CONCLUSION

Few studies have examined sensory processing patterns in school‐aged children born preterm according to ADHD status. Preterm children with ADHD exhibited a higher frequency of atypical sensory processing patterns and higher raw scores in specific sensory and behavioral sections of the SP‐2 than those without ADHD. These findings support the hypothesis that sensory processing difficulties may be more frequent among preterm children with ADHD and highlight the importance of considering sensory processing as part of the clinical assessment of this population. Further studies with larger cohorts are warranted to confirm these findings and to clarify the contributions of prematurity and ADHD to sensory processing outcomes.

Exploratory analyses identified an association between orotracheal intubation during the neonatal period and atypical scores in specific SP‐2 sections. However, this finding should be interpreted with caution, as it does not establish a causal relationship and may reflect the overall clinical complexity of the neonatal period rather than the isolated effect of orotracheal intubation.

Overall, these results suggest that sensory processing difficulties may be more common among preterm children with ADHD and underscore the importance of including sensory processing in the clinical assessment of this population. Further studies involving larger and more representative cohorts are needed to confirm these observations and to better understand the factors contributing to sensory processing outcomes in children born preterm.

## AUTHOR CONTRIBUTIONS

**Marina Estima Neiva Nunes**: Conceptualization; methodology; data curation; formal analysis; writing—original draft. **Carla Andrea Tanuri Cardoso Caldas**: Data curation; investigation; writing—review and editing. **Nelson Macedo Liporaci**: Formal analysis; statistical analysis; writing—review and editing. **Patricia Aparecida Zuanetti**: Investigation; data curation; writing—review and editing. **Angela Cristina Pontes Fernandes**: Investigation; data curation; writing—review and editing. **Carolina Augusta Portugal**: Investigation; data curation; writing—review and editing. **Ana Paula Andrade Hamad**: Conceptualization; supervision; writing—review and editing.

## CONFLICT OF INTEREST STATEMENT

The authors declare no conflicts of interest.

## ETHICS APPROVAL STATEMENT

The study was approved by the ethics committee under the number 43962621.9.0000.5440.

## PATIENT CONSENT STATEMENT

Parental written informed consent was obtained for all participants.

## CLINICAL TRIAL REGISTRATION

CAAE: 43962621.9.0000.5440.

## Data Availability

The data that support the findings of this study are available on request from the corresponding author. The data are not publicly available due to privacy or ethical restrictions.

## References

[1] Dunn W . Supporting children to participate successfully in everyday life by using sensory processing knowledge. Infants Young Child. 2007;20(2):84–101. 10.1097/01.IYC.0000264477.05076.5d

[2] Dunn W , Bennett D . Patterns of sensory processing in children with attention deficit hyperactivity disorder. OTJR: Occup Part Heal. 2002;22(1):4–15. 10.1177/153944920202200102

[3] Chen YC , Tsai WH , Ho CH , Wang HW , Wang LW , Wang LY , et al. Atypical sensory processing and its correlation with behavioral problems in late preterm children at age two. Int J Environ Res Public Health. 2021;18(12):6438. 10.3390/ijerph18126438 34198645 PMC8296277

[4] Little LM , Dean E , Tomchek S , Dunn W . Sensory processing patterns in autism, attention deficit hyperactivity disorder, and typical development. Phys Occup Ther Pediatr. 2018;38(3):243–254. 10.1080/01942638.2017.1390809 29240517

[5] Rani I , Agarwal V , Arya A , Mahour P . Sensory processing in children and adolescents with attention deficit hyperactivity disorder. J Atten Disord. 2023;27(2):145–151. 10.1177/10870547221129306 36239408

[6] Engel‐Yeger B , Ziv‐On D . The relationship between sensory processing difficulties and leisure activity preference of children with different types of ADHD. Res Dev Disabil. 2011;32(3):1154–1162. 10.1016/j.ridd.2011.01.008 21324640

[7] Delgado‐Lobete L , Pértega‐Díaz S , Santos‐del‐Riego S , Montes‐Montes R . Sensory processing patterns in developmental coordination disorder, attention deficit hyperactivity disorder and typical development. Res Dev Disabil. 2020;100:103608. 10.1016/j.ridd.2020.103608 32087509

[8] Shimizu VT , Bueno OFA , Miranda MC . Sensory processing abilities of children with ADHD. Braz J Phys Ther. 2014;18(4):343–352. 10.1590/bjpt-rbf.2014.0043 25076000 PMC4183255

[9] APA . Diagnostic and Statistical Manual of Mental Disorders: Fifth Edition Text Revision DSM‐5^TM^ . Washington: American Psychiatric Publishing; 2013.

[10] Dellapiazza F , Michelon C , Vernhet C , Muratori F , Blanc N , Picot MC , et al. Sensory processing related to attention in children with ASD, ADHD, or typical development: results from the ELENA cohort. Eur Child Adolesc Psychiatry. 2021;30(2):283–291. 10.1007/s00787-020-01516-5 32215734

[11] Mimouni‐Bloch A , Offek H , Rosenblum S , Posener I , Silman Z , Engel‐Yeger B . Association between sensory modulation and daily activity function of children with attention deficit/hyperactivity disorder and children with typical development. Res Dev Disabil. 2018;83:69–76. 10.1016/j.ridd.2018.08.002 30142575

[12] Jongmans M , Mercuri E , de Vries L , Dubowitz L , Henderson SE . Minor neurological signs and perceptual‐motor difficulties in prematurely born children. Arch Dis Child Fetal Neonatal Ed. 1997;76(1):F9–F14. 10.1136/fn.76.1.f9 9059179 PMC1720611

[13] Van Hus JWP , Jeukens‐Visser M , Koldewijn K , Van Sonderen L , Kok JH , Nollet F , et al. Comparing two motor assessment tools to evaluate neurobehavioral intervention effects in infants with very low birth weight at 1 year. Phys Ther. 2013;93(11):1475–1483. 10.2522/ptj.20120460 23766396

[14] Broström L , Vollmer B , Bolk J , Eklöf E , Ådén U . Minor neurological dysfunction and associations with motor function, general cognitive abilities, and behaviour in children born extremely preterm. Dev Med Child Neurol. 2018;60(8):826–832. 10.1111/dmcn.13738 29573402

[15] Buffone FRRC , Eickman SH , Lima MC . Processamento sensorial e desenvolvimento cognitivo de lactentes nascidos pré‐termo e a termo. Cadernos de Terapia Ocupacional da UFSCar. 2016;24(4):695–703. 10.4322/0104-4931.ctoAO0731

[16] Crozier SC , Goodson JZ , Mackay ML , Synnes AR , Grunau RE , Miller SP , et al. Sensory processing patterns in children born very preterm. Am J Occup Ther. 2016;70(1):7001220050p1–7001220057p1. 10.5014/ajot.2016.018747 26709425

[17] Pekçetin S , Sarıdaş B , Üstünyurt Z , Kayıhan H . Sensory‐processing patterns of preterm children at 6 years of age. Infants Young Child. 2019;32(1):33–42. 10.1097/IYC.0000000000000131

[18] Uusitalo K , Haataja L , Nyman A , Ripatti L , Huhtala M , Rautava P , et al. Preterm children's developmental coordination disorder, cognition and quality of life: a prospective cohort study. BMJ Paediatrics Open. 2020;4(1):e000633. 10.1136/bmjpo-2019-000633 32518843 PMC7254160

[19] Bhutta AT , Cleves MA , Casey PH , Cradock MM , Anand KJ . Cognitive and behavioral outcomes of school‐aged children who were born preterm: a meta‐analysis. JAMA. 2002;288(6):728–737. 10.1001/jama.288.6.728 12169077

[20] Machado ACCP , Oliveira SR , Magalhães LC , Miranda DM , Bouzada MCF . Processamento sensorial no período da infância em crianças nascidas pré‐termo: revisão sistemática. Rev Paul Pediatr. 2017;35(1):92–101. 10.1590/1984-0462/;2017;35;1;00008 28977307 PMC5417800

[21] Fabio RA , Orsino C , Lecciso F , Levante A , Suriano R . Atypical sensory processing in adolescents with attention deficit hyperactivity disorder: a comparative study. Res Dev Disabil. 2024;146:104674. 10.1016/j.ridd.2024.104674 38306842

[22] Bröring T , Königs M , Oostrom KJ , Lafeber HN , Brugman A , Oosterlaan J . Sensory processing difficulties in school‐age children born very preterm: an exploratory study. Early Hum Dev. 2018;117:22–31. 10.1016/j.earlhumdev.2017.12.003 29227903

[23] Chorna O , Solomon JE , Slaughter JC , Stark AR , Maitre NL . Abnormal sensory reactivity in preterm infants during the first year correlates with adverse neurodevelopmental outcomes at 2 years of age. Arch Dis Child Fetal Neonatal Ed. 2014;99(6):F475–F479. 10.1136/archdischild-2014-306486 25053637 PMC4783156

[24] Mitchell AW , Moore EM , Roberts EJ , Hachtel KW , Brown MS . Sensory processing disorder in children ages birth–3 years born prematurely: a systematic review. Am J Occup Ther. 2015;69(1):6901220030p1–6901220030p11. 10.5014/ajot.2015.013755 25553748

[25] Niutanen U , Harra T , Lano A , Metsäranta M . Systematic review of sensory processing in preterm children reveals abnormal sensory modulation, somatosensory processing and sensory‐based motor processing. Acta Paediatr (Stockholm). 2020;109(1):45–55. 10.1111/apa.14953 31350861

[26] Philpott‐Robinson K , Lane SJ , Korostenski L , Lane AE . The impact of the neonatal intensive care unit on sensory and developmental outcomes in infants born preterm: a scoping review. Br J Occup Ther. 2017;80(8):459–469. 10.1177/0308022617709761

[27] Wickremasinghe AC , Rogers EE , Johnson BC , Shen A , Barkovich AJ , Marco EJ . Children born prematurely have atypical Sensory Profiles. J Perinatol. 2013;33(8):631–635. 10.1038/jp.2013.12 23412641 PMC3738436

[28] Pfeiffer B , Daly BP , Nicholls EG , Gullo DF . Assessing sensory processing problems in children with and without attention deficit hyperactivity disorder. Phys Occup Ther Pediatr. 2015;35(1):1–12. 10.3109/01942638.2014.904471 24712841

[29] Schulz SE , Kelley E , Anagnostou E , Nicolson R , Georgiades S , Crosbie J , et al. Sensory processing patterns predict problem behaviours in autism spectrum disorder and attention‐deficit/hyperactivity disorder. Adv Neurodev Disord. 2023;7(1):46–58. 10.1007/s41252-022-00269-3

[30] Shimizu VT , Miranda MC . Processamento sensorial na criança com TDAH: uma revisão da literatura. Revista Psicopedagogia. 2012;29(89):256–268.

[31] Dean E , Dunn W , Little LM . Validity of the Sensory Profile 2: a confirmatory factor analysis. Am J Occup Ther. 2016;70(1_Suppl):S242.

[32] Dunn W . Perfil Sensorial 2: Manual do Usuário. São Paulo: Pearson Clinical Brasil; 2017.

[33] SciELO ‐ Brasil ‐ Apresentação de uma versão em português para uso no Brasil do instrumento MTA‐SNAP‐IV de avaliação de sintomas de transtorno do déficit de atenção/hiperatividade e sintomas de transtorno desafiador e de oposição Apresentação de uma versão em português para uso no Brasil do instrumento MTA‐SNAP‐IV de avaliação de sintomas de transtorno do déficit de atenção/hiperatividade e sintomas de transtorno desafiador e de oposição [Internet]. [citado 12 de julho de 2024]. Disponível em: https://www.scielo.br/j/rprs/a/SQPJkswbm5FWM6kSzm6SQkG/

[34] Achenbach TM , Rescorla L . Manual for the ASEBA school‐age forms & profiles: an integrated system of multiinformant assessment, ASEBA; 2001.

[35] ABEP. Critério Padrão de Classificação Econômica Brasil [Internet]. 2021 [citado 1° de junho de 2021]. Disponível em: https://www.abep.org/criterio-brasil

[36] Mattos P , Serra‐Pinheiro MA , Rohde LA , Pinto D . A Brazilian version of the MTA‐SNAP‐IV for evaluation of symptoms of attention‐deficit/hyperactivity disorder and oppositional‐defiant disorder. Revista de Psiquiatria do Rio Grande do Sul 2006;28(3):290–297. 10.1590/S0101-81082006000300008

[37] Lickliter R . The integrated development of sensory organization. Clin Perinatol. 2011;38(4):591–603. 10.1016/j.clp.2011.08.007 22107892 PMC3223372

[38] Eeles AL , Anderson PJ , Brown NC , Lee KJ , Boyd RN , Spittle AJ , et al. Sensory profiles obtained from parental reports correlate with independent assessments of development in very preterm children at 2years of age. Early Hum Dev. 2013;89(12):1075–1080. 10.1016/j.earlhumdev.2013.07.027 23978398

[39] Rubia K . Cognitive neuroscience of attention deficit hyperactivity disorder (ADHD) and its clinical translation. Front Hum Neurosci. 2018;12:100. 10.3389/fnhum.2018.00100 29651240 PMC5884954

[40] Yochman A , Parush S , Ornoy A . Responses of preschool children with and without ADHD to sensory events in daily life. Am J Occup Ther. 2004;58(3):294–302. 10.5014/ajot.58.3.294 15202627

[41] Tomasi D , Volkow ND . Abnormal functional connectivity in children with attention‐deficit/hyperactivity disorder. Biol Psychiatry. 2012;71(5):443–450. 10.1016/j.biopsych.2011.11.003 22153589 PMC3479644

